# Structural, Morphological, and Antibacterial Attributes of Graphene Oxide Prepared by Hummers’ and Brodie’s Methods

**DOI:** 10.3390/molecules30020240

**Published:** 2025-01-09

**Authors:** Vittorio Marsala, Yuriy Gerasymchuk, Maria Luisa Saladino, Emil Paluch, Magdalena Wawrzyńska, Vitalii Boiko, Xiang Li, Cristina Giordano, Dariusz Hreniak, Beata Sobieszczańska

**Affiliations:** 1 Biological, Chemical, and Pharmaceutical Science and Technology Department–STEBICEF, University of Palermo, 90128 Palermo, Italy; vittorio.marsala@unamur.be; 2 Division of Optical Spectroscopy, Institute of Low Temperature and Structure Research, Polish Academy of Sciences, 50-422 Wroclaw, Poland; y.gerasymchuk@intibs.pl (Y.G.); v.boiko@intibs.pl (V.B.); d.hreniak@intibs.pl (D.H.); 3 Unit of Nanomaterials Chemistry, Department of Chemistry, Namur Institute of Structured Matter (NISM), University of Namur, 5000 Namur, Belgium; 4 Consiglio Nazionale delle Ricerche, Istituto per i Processi Chimico Fisici, 98158 Messina, Italy; 5 Department of Preclinical Studies, Faculty of Health Sciences, Wroclaw Medical University, 50-367 Wroclaw, Poland; emil.paluch@umw.edu.pl (E.P.); magdalena.wawrzynska@umw.edu.pl (M.W.); 6 Department of Microbiology, Wroclaw Medical University, 50-368 Wroclaw, Poland; beata.sobieszczanska@umw.edu.pl; 7 Institute of Physics of the National Academy of Science of Ukraine, Prospect Nauky 46, UA-03028 Kyiv, Ukraine; 8 School of Physical and Chemical Sciences, Queen Mary University of London, Mile End Road, London E1 4NS, UK; btx837@qmul.ac.uk (X.L.); c.giordano@qmul.ac.uk (C.G.)

**Keywords:** graphene oxide, aqueous nanodispersions, antibacterial activity, biofilm

## Abstract

Graphite oxidation to graphene oxide (GO) is carried out using methods developed by Brodie (GO-B) and Hummers (GO-H). However, a comparison of the antibacterial properties based on the physicochemical properties has not been performed. Therefore, this paper outlines a comparative analysis of GO-H and GO-B on antibacterial efficacy against Gram-positive and Gram-negative bacterial cultures and biofilms in an aqueous environment and discusses which of the properties of these GO nanomaterials have the most significant impact on the antibacterial activity of these materials. Synthesis of GO with Brodie’s and modified Hummers’ methods was followed by an evaluation of their structural, morphological, and physicochemical properties by Raman, FTIR, UV–vis spectroscopy, and X-ray diffraction (XRD). The GO-B surface appeared more oxidized than that of GO-H, which could be crucial for interactions with bacteria. According to our results, GO-B demonstrated notably superior anti-biofilm efficacy. Despite its higher production cost, GO-B exhibits more excellent capabilities in combating bacterial biofilms than GO-H.

## 1. Introduction

Graphene oxide (GO) is the oxidized form of graphite, a two-dimensional layer of carbon atoms with sp^2^ hybridization organized into a honeycomb multilayered lattice structure [1]. GO has hydrophilic properties due to carbonyl, hydroxyl, and epoxy functional groups on its surface; hence, it disperses in an aqueous environment, forming stable colloids [2,3]. It is essential to highlight that GO is a non-stoichiometric compound of carbon, oxygen, and hydrogen in various ratios that depend on the synthetic methodology. Therefore, the method used to synthesize this interesting material strongly affects the final result as it is pivotal in determining the amount of oxygen-containing functional groups, such as carboxyl (-COOH), ketone (=O), and hydroxyl (-OH) groups [4]. As a matter of fact, all currently used GO production techniques are derived from Brodie’s method, which was first described in 1859 and then improved by Staudenmaier in 1898 [5,6]. Based on Brodie’s process, which used potassium chlorate added to a slurry of graphite in fuming nitric acid to oxidize graphite, in 1898, Staudenmaier improved the technique by replacing most of the nitric acid with concentrated sulfuric acid [6]. Nowadays, however, the most common method of graphite oxidation used is the alternate Hummers’ and Offeman’s oxidation method, now called Hummers’ technique, which allows the oxidization of graphite into GO within a few hours using sodium nitrate and potassium permanganate dissolved in concentrated sulfuric acid [7]. Although both Brodie’s and Hummers’ methods ultimately produce graphene oxide (GO), the fundamental differences in the oxidation process of graphite significantly influence its physicochemical properties [8]. The cost of reagents for GO production by Brodie’s method (GO-B) significantly exceeds that of GO obtained by Hummers’ technique (GO-H). Additionally, Brodie’s technique of GO obtainment is more time-consuming, challenging, and hazardous than the modified Hummers’ method [9,10]. The improved Hummers’ method has the lowest toxicity and there are several advantages to the synthesized product, justifying its common usage [9,11]. According to a study by Lavin-Lopez et al. [11], alternate Hummers’ method, compared to Brodie’s technique, gives the best process yield and is environmentally friendly because there are no toxic gases generated during the preparation. Moreover, GO-H has a more organized structure compared to GO-B.

On the other hand, many other studies point to the superiority of GO-B over GO-H. Pedrosa et al. [12] demonstrated that GO-B had better photocatalytic properties than GO-H. Talyzin et al. [13], comparing GO-H and GO-B, revealed a higher number of carbonyl and carboxyl groups in GO-H than in GO-B, resulting in defects of the graphene “skeleton”, i.e., holes, and overall disruption of the carbon–carbon bond network, more substantial deviation from planar flake shape, and poor ordering of the graphene oxide layers. Their results also suggest that functional groups in GO-H are less homogeneously distributed over the flake surface, forming some nanometer-sized graphene areas, in contrast with GO-B.

Besides affecting physicochemical characteristics, the graphite oxidation technique also impacts its antibacterial activity. In general, the antimicrobial efficacy of GO occurs through its various physical and chemical interactions with microorganisms, including bacterial cell damage caused by the extremely sharp edges of the GO nanowalls [14,15], the extraction of phospholipids [16], the wrapping of bacterial cells by the large surface area of GO sheets [17], reactive oxygen species (ROS) generation leading to genomic DNA, and protein and cellular metabolism disruption [18,19,20]. Direct physical contact with sharp GO nanosheet edges (nano-blades) mainly affects Gram-positive bacteria with a thick peptidoglycan layer on their surface. In contrast, the outermost outer membrane of Gram-negative bacteria seems more resistant to the GO nano-blade’s antibacterial activity, a fact which can be associated with its fluidity [15]. On the other hand, Tu et al. [16] demonstrated that a GO nanosheet could extract large amounts of phospholipids from the cell membranes of Gram-negative *Escherichia coli* (*E. coli*) cells because of the strong dispersion interactions between GO and lipid molecules, thus demonstrating that it exhibits bactericidal activity against Gram-negative bacteria. As mentioned earlier, the antibacterial activity of GO nanomaterials largely depends on their physicochemical characteristics, e.g., lateral size, purity, charge, functional groups, degree of oxidation, and hydrophilicity [21]. Perreault et al. [21] revealed that decreasing the GO nanosheet area from 0.65 to 0.01 µm^2^ increased its antibacterial activity fourfold. This was attributed to the oxidative mechanism associated with the higher damage density in bacterial cell walls caused by miniature GO sheets. On the other hand, the bacterial cell-wrapping mechanism by GO sheets, which limits nutrient availability and, thus, has mostly bacteriostatic activity, disappeared for 0.65 µm^2^ sheets, indicating that the GO nanosheet area is essential for GO’s antibacterial activity.

Considering the physicochemical differences revealed in many studies between GO obtained by Brodie’s and Hummers’ methods, GO-H and GO-B may exhibit different antibacterial activity. However, to our knowledge, the antibacterial efficacy of GO-H and GO-B has yet to be compared, which has provided the basis for our study. Therefore, in this work, we report the preparation, structural analysis, and antibacterial characterization of two GO samples obtained with these two conventional methods, i.e., Brodie’s and Hummers’, to evaluate if the method can affect the antibacterial activity. The antibacterial activity of the GOs (minimal inhibitory and bactericidal values) was examined against planktonic Gram-positive and Gram-negative bacteria. Moreover, GO-H and GO-B’s influence on bacterial biofilm formation and eradication was assessed.

## 2. Results

### 2.1. Structural and Morphological Characterization of GO Samples

**Figure 1:**
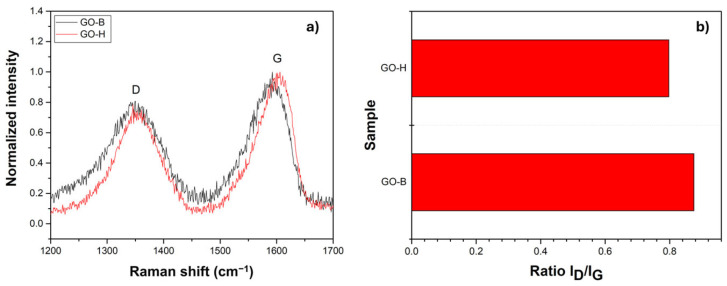
(**a**) Raman spectra of GO-B and GO-H samples and (**b**) ratio between the intensity of the D band and G band in the samples.

The GO obtained by the modified Hummers’ and Brodie’s methods were investigated to evaluate their composition, biocompatibility, and antibacterial properties. As reported in Figure 1a, the Raman spectra of GO-H and GO-B exhibit two broad bands in the 1200–1800 cm^−^^1^ range, a typical profile of graphene-based nanomaterials. The D band at 1350 cm^−^^1^ is defect-related, and it can be attributed to carbon–oxygen bonds formed during graphite oxidation, grain boundaries, and point defects, which can cause a deviation from the ideal planar graphene structure [22]. The G band, centered at 1580 cm^−^^1^, originated from the plane vibrations of carbon rings [23]. The broadened G and D bands indicate a severe disruption of the sp^2^ carbon lattice. In both samples, no significant differences between the two bands are present, even if a small shift (6 cm^−^^1^) between the two samples can be observed. The observed shift could be due to several factors, such as the presence of defects or deformations in the graphene structure as well as strain on the structure due to the presence of hydroxyl, epoxide, carboxyl, and carbonyl groups. The I_D_/I_G_ ratio is related to the sp^3^/sp^2^ carbon ratio, and it is important for understanding the antibacterial activity of graphene oxide as a higher ratio suggests a greater density of defects, which may contribute to the material’s bactericidal effect [24]. The I_D_/I_G_ ratio for GO-H and GO-B reported in Figure 1b shows that the GO-H has a lower I_D_/I_G_ ratio than the GO-B, suggesting that the latter’s structure is less organized.

**Figure 2:**
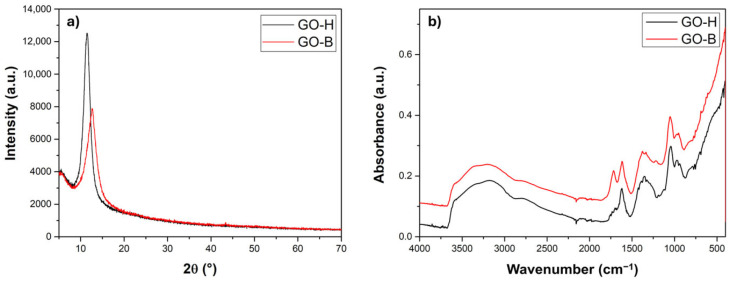
(**a**) XRD patterns and (**b**) FTIR spectra of GO-B and GO-H samples.

The XRD patterns of GO-H and GO-B were obtained from the freeze-dried aqueous dispersions, and are reported in Figure 2. The XRD analysis revealed the presence of a sharp peak at 2θ = 11.5° and 12.7° for the GO-H and the GO-B, respectively. These peaks are related to an interlayer spacing of 0.77 nm for GO-H and 0.70 nm for GO-B, respectively. Moreover, the absence of a peak around 2θ = 26.5° confirms the loss of the starting graphite’s major part of the sp^2^ structure, in agreement with Raman spectra. The difference in the interlayer distance could be attributed to the different levels of oxygen-containing groups. The obtained GO was characterized by Fourier transform infrared spectroscopy (FT-IR) and X-ray photoelectron spectroscopy (XPS) to investigate the latter hypothesis further.

FT-IR spectra show the characteristic bands of GO, as reported by Emiru et al. [25]. However, the bands of carboxyl and carbonyl groups are present in the GO-B spectra and are absent in the GO-H spectra. In addition, the stretching bands of -C=O,-C-OH, and -COOH groups, are present in the GO-B spectra (Table 1). These findings allow us to state that the GO-B surface is more oxidized than that of GO-H, which could be crucial in the case of interactions with bacteria.

**Figure 3:**
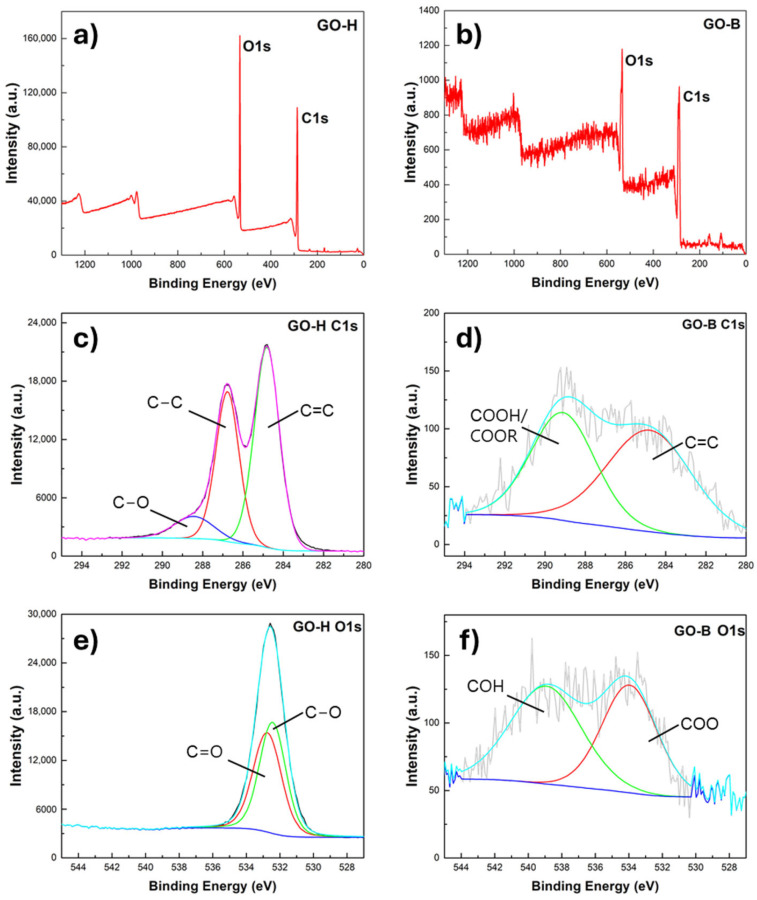
XPS survey (**a**,**b**), C1s (**c**,**d**), and O1s (**e**,**f**) spectra of GO-H and GO-B samples.

XPS spectra are reported in Figure 3. From survey spectra, we can observe that the samples are mainly composed of C and O. GO-H shows the C peaks (Figure 3c) at around 284.8, 286.7, and 288.4 eV. The peak located at 284.8 eV was assigned to the sp^2^ C=C band of graphitized carbon, while the peaks at around 286.7 and 288.4 were attributed to the C-C sp^3^ bonds in the amorphous phase and C-O bands, respectively. High-resolution O1s spectra (Figure 3e) show peaks at 532.7 eV (49.12 wt.%) and 532.4 eV (50.88 wt.%), which can be assigned to C-O/C=O. In GO-B, high-resolution C1s spectra (Figure 3d) show peaks at 284.8 eV and 289.1 eV, and they are attributed to the C=C bond with a content of 55.56 wt.% and the COOH/COOR bond with a content of 44.44 wt.%. O1s spectra (Figure 3f) show peaks at 533.9 eV and 538.92 eV, which are assigned to COO and COH, respectively. So, the different procedures generate different organic moieties.

**Figure 4:**
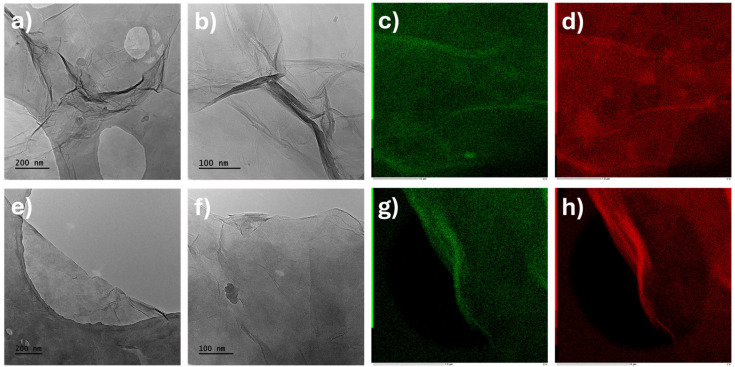
TEM micrographs of (**a**,**b**) GO-H and (**e**,**f**) GO-B samples and EDS maps of oxygen (red) and carbon (green) of (**c**,**d**) GO-H and (**g**,**h**) GO-B samples.

The samples were also characterized by Transmission Electron Microscopy (TEM) to evaluate their differences in terms of size and aggregation. The TEM micrographs are reported in Figure 4. TEM analysis of GO dispersions shows that the GO sheets consist of a few stacked layers. Dark areas indicate the thick stacking nanosheets of GO layers with some oxygen functional groups, while the transparency areas suggest that the graphene oxide sheets have a few layers resulting from exfoliation. All TEM images show clear folding, indicating the formation of oxygen functional groups, as confirmed by EDS mapping (Figure 4c,d,g,h), FT-IR (Figure 2b), and XPS (Figure 3) data.

**Figure 5:**
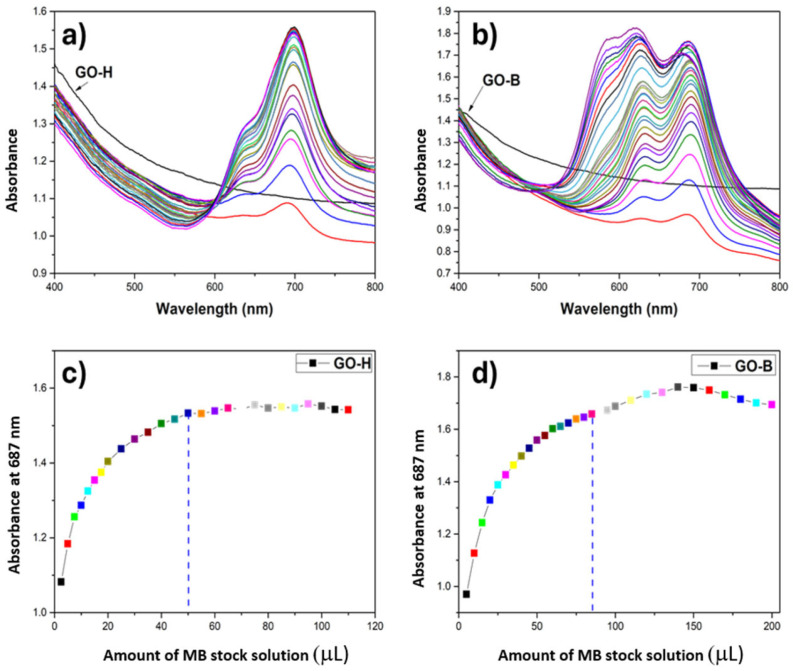
Graphs of the results of methylene blue titration of GO-H (**a**) and GO-B (**b**) samples and graphs of the dependence of absorbance intensity on the amount of added MB stock solution for GO-H (**c**) and GO-B (**d**).

UV–vis spectra, reported in Figure 5, were acquired to evaluate the surface area of GO-H and GO-B samples. In our case, the point corresponding to the maximum coverage of the material surface with the dye is the point on the graph of the dependence of absorbance intensity on the concentration of methylene blue in the cuvette at which the increase in absorbance intensity stops or decreases.

Surface area measurements were achieved, starting from an aqueous dispersion of known GO composite concentration, and increased amounts of MB were successfully added. The evolution of spectra was recorded. By tracking the spectra, we reached the point that had different trends, which led us to understand the saturation point in each experiment. The area covered by mg of MB was estimated at 2.54 m^2^ for diluted GO suspensions. The surface area values are obtained by considering that point. According to our calculations, GO-H has a lower surface area (about 700 m^2^/g) than the graphite oxide obtained by Brodie’s method. The GO-B has a higher surface area, about seven times more (1000 m^2^/g) than the material obtained by Hummers’ method. The larger specific surface area of GO-B compared to GO-H may influence the intensity of the material’s interaction with bacteria and, thus, affect the antibacterial activity of the investigated materials.

### 2.2. Antibacterial Activity of GO-B and GO-H Samples

The minimal bactericidal concentration (MBC) and minimal inhibitory concentration (MIC) values of GO-B and GO-H for two Gram-positive and two Gram-negative bacterial strains in an aqueous environment were evaluated. GO-B and GO-H demonstrated antibacterial (MBC) activity at 100 µg/mL against Gram-negative species represented by *Pseudomonas aeruginosa* ATCC 27853 and *E. coli* ATCC 25922 strains. On the contrary, MBC values of GO-B and GO-H for Gram-positive species represented in the study by *Staphylococcus aureus* ATCC 25923 and *Enterococcus faecalis* ATCC29212 reference strains were much higher, i.e., ≥1 mg/mL (Table 2). The MIC values of GO-B and GO-H for Gram-negative bacterial species were similar. They amounted to 30 µg/mL, and for Gram-positive species, 60 µg/mL (Table 2). Our results follow other studies reporting higher antibacterial activity of GO against Gram-negative bacteria than Gram-positive ones [14,19,26,27,28]. Pulingam et al. [26] demonstrated on GO-H that bacterial cell entrapment was the primary antibacterial mechanism of GO towards Gram-positive bacteria like *S. aureus* and *E. faecalis*. In contrast, membrane damage via the direct physical contact of GO nanoparticles working as nano-blades was prevalent antibacterial activity against Gram-negative species like *E. coli* and *P. aeruginosa*. Similarly, Farid et al. [29] demonstrated on *E. coli* and *S. aureus* strains that commercially available GO nanosheets with oxidized edges mechanically disrupted bacterial cell membranes and induced the production of reactive oxygen species (ROS) in bacteria. Moreover, the GO nanosheets inhibited biofilm formation by these bacterial strains [29].

### 2.3. The Impact of GO-H and GO-B on Biofilm Production

After 48 h of incubation, all tested bacterial strains produced biofilms on glass slides. Biofilms produced without GO consisted mainly of viable bacteria and small portions of dead ones determined by LIVE/DEAD fluorescence staining. The bacteria killed in biofilms ranged from 1.3% of total biofilm mass for *S. aureus* (through 5.4% for *E. coli* and 2.7% for *P. aeruginosa*) to 8.1% for *E. coli*.

**Figure 6:**
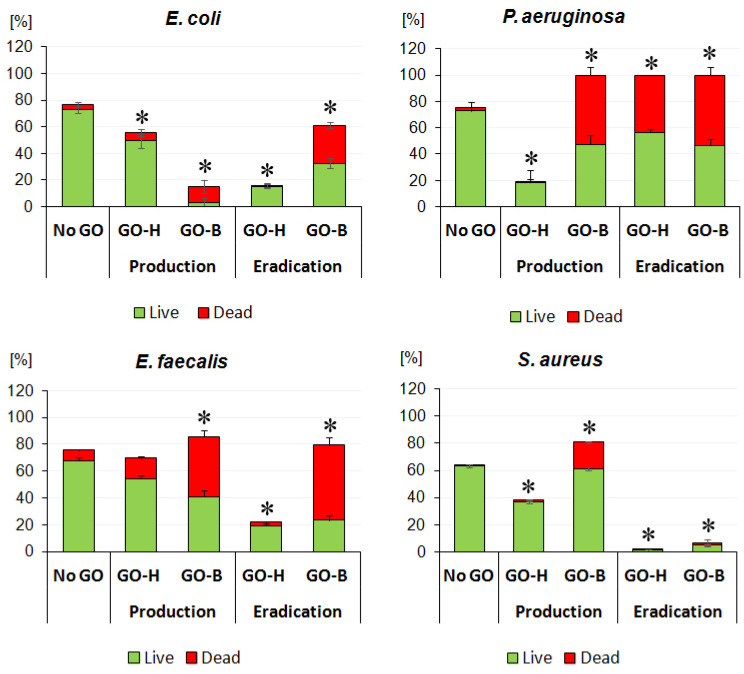
The impact of GO-B and GO-H on bacterial biofilm production and eradication. * indicates statistical differences compared to negative control (No GO) calculated by the ANOVA test with *p* < 0.05 considered significant.

**Figure 7:**
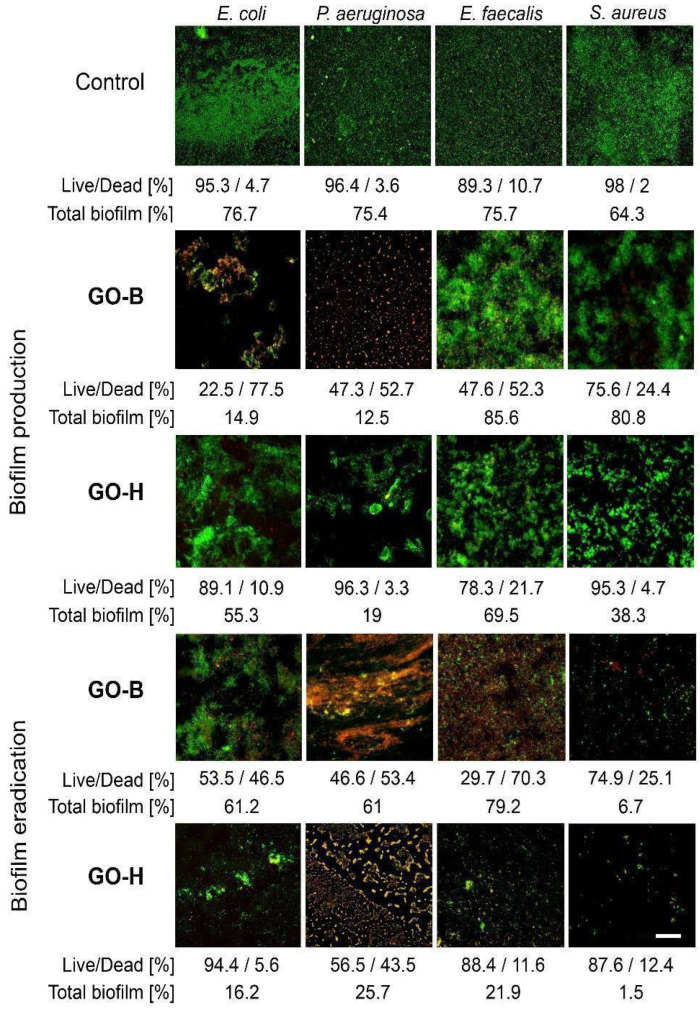
Effect of GO on the formation and eradication of bacterial biofilms. The white scale bar in the lower right image indicates a magnification of 400× for all images.

The ability of biofilm formation assessed after 48 h indicated that GO-B at 60 µg/mL inhibited biofilm formation by *P. aeruginosa* and *E. coli* by >80% (*p* < 0.05). Moreover, 77.5% of *E. coli* and 52.7% of *P. aeruginosa* cells within biofilms were dead compared to biofilms without GO (*p* < 0.05). Although, at twice the strength concentrations, the GO-B inhibitory effect on biofilm formation was less pronounced for Gram-positive bacteria, where the total biofilms produced by these bacteria were even higher in GO-B than in those without (Figure 6). However, about 52.3% (*p* < 0.05) of *E. faecalis* cells within the GO-B biofilm were dead compared to those within the control biofilm. In the case of *S. aureus*, only 24.4% (*p* > 0.05) of cells within the GO-B biofilm were dead. In contrast, biofilms produced in the presence of GO-H, although reduced by 44.7% and 81% for *E. coli* and *P. aeruginosa*, mainly consisted of live bacterial cells. Similarly, biofilms produced by *S. aureus* and *E. faecalis* in the presence of GO-H were reduced by 26% (*p* < 0.05) and 6.2% (*p* > 0.05), respectively. Moreover, these biofilms were also mainly alive. The percentage of dead bacteria in both GO-H biofilms for *S. aureus* and *E. faecalis* was 4.7% and 21.7% (*p* > 0.05), respectively (Figure 7). These results indicate that GO-B at concentrations corresponding to double MIC value inhibits biofilm formation by Gram-negative bacteria much better than GO-H and kills Gram-positive bacteria within biofilms.

### 2.4. Biofilm Eradication in the Presence of GO-B and GO-H

Evaluation of the effect of GO-B and GO-H on biofilm eradication showed that GO-B reduced mature *E. coli* and *P. aeruginosa* biofilms by only 20.2% and 10.1% (*p* > 0.05), respectively (Figure 6). On the contrary, in the presence of GO-B, *S. aureus* biofilm was reduced by 89.6% (*p* < 0.05), and its viability decreased by 25.1% (*p* < 0.05). Interestingly, GO-B did not affect the total mass of the mature *E. faecalis* biofilm but significantly reduced its viability by 70.5% (*p* < 0.05). A similar decrease in bacteria viability in GO-B was observed in *E. coli* and *P. aeruginosa* biofilms, i.e., by 46.5% and 53.4% (*p* < 0.05), respectively. On the other hand, GO-H showed a significantly better effect than GO-B in eradicating mature biofilm biomass. For *E. coli*, *P. aeruginosa*, *S. aureus*, and *E. faecalis* biofilms, GO-H reduced the biomass of biofilms by 78.9%, 65.9%, 97.7%, and 71% (*p* < 0.05), respectively (Figure 7). Nevertheless, only in the case of *P. aeruginosa* GO-H reduced the viability of the biofilm by 43.5% (*p* < 0.05). For other biofilms, the decrease in viability ranged from 5.6% for *E. coli* biofilm to <13% for *S. aureus* and *E. faecalis* biofilms (*p* > 0.05). These results indicated that both GOs showed excellent anti-biofilm activity at different stages. GO-B inhibited the growth of biofilms more effectively than GO-H, while GO-H reduced the total biomass of biofilms significantly better than GO-B.

## 3. Discussion

GO can physically impact bacteria through the direct contact of GO flakes with bacterial cells or have a chemical effect by the interaction of GO’s active chemical groups with the bacteria’s surface structures [30]. Thus, the antibacterial activity of GO depends on its physicochemical characteristics, e.g., lateral size, purity, charge, functional groups, degree of oxidation, and hydrophilicity, which results from its preparation technique [19,20]. However, the divergent results of studies on the antibacterial activity of GO flakes presented in the literature indicate that the obtained GO preparations may differ in their physicochemical properties, which ultimately translates into their effect on Gram-positive and Gram-negative bacteria.

According to some researchers [31,32,33], wrapping bacterial cells with GO flakes is the primary antibacterial mechanism against Gram-negative bacteria, which present an outer membrane on their surface. Direct contact of the outer membrane with GO flakes may lead to its destruction (bactericidal effect). On the other hand, the wrapping of bacterial cells by GO flakes is also indicated by researchers as a mechanism of the antibacterial activity of GO against Gram-positive bacteria, which present a thick layer of rigid peptidoglycan on their surface [15,26]. Due to this specific structure of the cell wall of Gram-positive bacteria, wrapping bacterial cells seems to have little effect on their viability. However, GO flakes’ sharp edges can penetrate the thick peptidoglycan layer, destroying its structure and ultimately disrupting bacterial cells due to cytoplasm leakage [26]. The effect of the sharp nano-edges of GO flakes is also effective in the case of Gram-negative bacteria. Akhavan et al. [15] measured the efflux of cytoplasmic materials of the bacteria treated with GO and demonstrated that the cell membrane damage was caused by direct contact with the extremely sharp edges of the nanowalls. In their study, the Gram-negative *E. coli* with an outer membrane were more resistant to the cell membrane damage caused by the nanowalls than the Gram-positive *S. aureus* lacking the outer membrane.

Although GO-B and GO-H’s physicochemical properties differed in our study, their antibacterial inhibitory activity, measured as MIC concentration for Gram-positive and Gram-negative bacteria, was similar. However, for Gram-negative bacteria, they were one order of magnitude higher than for Gram-positive. On the contrary, MBC values for Gram-positive bacteria were higher or equal to the initial concentration of both GO solutions, indicating a lack of bactericidal activity for *S. aureus* and *E. faecalis*. MBC values for Gram-negative bacteria were ten times lower than for Gram-positive species, indicating significantly better GO-B and GO-H bactericidal activity against this group of bacteria. In contrast to our results, Sengupta et al. [33] analyzed the antibacterial activity of GO at 3 mg/mL against *S. aureus* and observed growth inhibition of 93.7% after 5 h of incubation. However, it is unclear whether the GO killed these bacteria and demonstrated bactericidal activity or inhibited their growth compared to the negative control (bacteriostatic activity). Moreover, in their study, the obtained GO, at the same concentration, had significantly less impact on *P. aeruginosa*. In turn, Olczak et al. [34], using an aqueous dispersion of commercial GO (1 mg/mL) by flow cytometry analysis, demonstrated the best bactericidal activity of the GO after 30 min of incubation with *S. aureus*, *Streptococcus mutans*, *E. faecalis,* and *E. coli*. The bactericidal activity of GO they tested was 50 µg/mL against *E. coli*, thus, half as much as in our study. However, in their study, the GO concentrations cidal to Gram-positive species tested ranged from 100 µg/mL to 200 µg/mL. This confirms our results, indicating higher resistance of Gram-positive bacteria to GO. Similarly, Nanda et al. [32] established MIC values of GO-H for *E. coli* at 1 µg/mL but for *E. faecalis* at 4 µg/mL.

When delving into the literature on the antibacterial activity of GO, primarily obtained by Hummers’ method, it becomes apparent that each study employs a unique combination of GO’s incubation times with bacteria, methods of assessing its effect, and initial concentrations of GO [23,35,36,37]. This wide array of approaches, often presenting results as percentages of live or killed bacteria, underscores the lack of standardization and the difficulty in comparing research results. Nevertheless, most GO-H studies indicate that it has better activity against Gram-negative than Gram-positive bacteria, which is also true of our results.

Bacterial biofilms are intricate structures that bacteria produce in natural environments and the human body. Unfortunately, these structures, where bacteria are embedded in a layer of exopolysaccharide, are also linked to numerous human infections. Due to the protective exopolysaccharide layer, biofilm resilience makes them difficult to eradicate. Biofilms’ resistance to antibiotics is primarily due to the exopolysaccharide layer, which hinders the penetration of antibiotics and shields the biofilm from phagocytosis [38]. The involvement of biofilms in difficult-to-treat infections drives the search for antibacterial agents that penetrate biofilms and enable their eradication or prevent their formation. Hence, the study compared GO-B and GO-H activity against biofilm formation and biofilm eradication efficacy.

In our studies, biofilms formed by the studied bacterial species were assessed on collagen-coated slides to bring the research model closer to in vivo conditions, e.g., wounds, in which proteins can protect adhering bacteria. GO-B again demonstrated a significantly better ability to inhibit biofilm formation in vitro by Gram-negative bacteria but also considerably reduced their viability within the biofilm compared to GO-H. In the case of Gram-positive bacteria, although both graphene oxides, i.e., GO-B and GO-H, reduced their ability to form biofilms, they did not have as significant an effect on reducing their viability as in the case of Gram-negative bacteria. We obtained similar results for both GO in eradicating biofilms formed by Gram-negative and Gram-positive bacteria. GO-B reduced mature biofilms formed by Gram-negative bacteria better than GO-H. In turn, the effect of GO-B on the total biofilm mass was smaller in Gram-positive bacteria. Still, in this case, an apparent bactericidal effect of GO-B was noted, which was more potent than in the case of GO-H. Di Giulio et al. [39] studied the impact of commercially available GO on the production and eradication of biofilms of Gram-positive (*S. aureus*) and Gram-negative (*P. aeruginosa*) bacteria at a concentration of 50 µg/mL and showed that the tested GO significantly reduced the formation of biofilms by both bacterial species by wrapping bacterial cells. These investigators subsequently confirmed the anti-biofilm activity of GO in vitro using the multispecies Lubbock chronic wound biofilm model [39]. Similarly, Saeed et al. [40] demonstrated a significant disruption of *S. aureus* biofilm structure by commercially available GO at 100 µg/mL.

Considering our research results, which distinctly indicated better antibacterial activity of GO-B than GO-H, we analyzed the physicochemical properties of both graphene oxides, which could be responsible for these differences.

The GO-B nanosheet was characterized by a much more significant amount of oxygen in the form of singlet oxygen, carboxyl, aldehyde, hydroxyl, and epoxy groups on the edges, which contributed to their delamination and affected the sharpness of the edges. Additionally, the presence of carboxyl groups increased the acidification of the environment.

One of GO’s most essential antibacterial activities is the oxidative stress induced by its active oxygen groups. Oxidative stress plays a primary role in GO’s antibacterial activity, especially against bacteria growing on GO coatings, where the cell-wrapping and nano-edge mechanisms generally do not matter [41]. Exposure of bacterial cells to reactive oxygen species (ROS) damages cellular components, including DNA, membrane lipids, and proteins [42]. Hence, GO bactericidal activity, to a large extent, relies on the induction of oxidative stress in bacterial cells. On the other hand, bacteria have developed numerous defense mechanisms to eliminate the impact of ROS, such as the production of enzymes that convert toxic ROS into harmless compounds before they cause damage to the cell. This role is played by catalase in Gram-positive bacteria, while in Gram-negative bacteria, it is catalase and superoxide dismutase [43].

Based on their study, Liu et al. [44] proposed that GO induces superoxide anion-independent oxidation in bacteria by ensuing glutathione oxidation, a mediator of bacterial redox state. Glutathione is one of the most abundant thiols present in proteobacteria. In addition to its key role in maintaining the proper oxidation state of protein thiols, glutathione also serves a key function in protecting the cell from the action of low pH, chlorine compounds, and oxidative and osmotic stresses [45]. This may explain the higher activity of GO against Gram-negative bacteria, which belong to Proteobacteria, than against Gram-positive bacteria, as reported by most researchers. In turn, Ravikumar et al. [46] demonstrated that in Gram-positive bacteria, i.e., *S. aureus*, GO treatment generated intracellular ROS, which correlated to the loss of cell viability. Their proteomics analysis revealed alteration in the expression of the cell membrane, oxidative stress response, general stress response, and virulence-associated proteins in GO-treated bacterial cells. It is, therefore, possible that GO chemically affects Gram-positive and Gram-negative bacteria differently. Furthermore, many researchers have emphasized that the loss of bacterial viability increases with the GO concentration and the time of bacterial exposure to GO [18,34,46].

Moreover, Brodie’s method allowed for obtaining much smaller GO nanosheets than Hummers’. Although it is commonly assumed that the sharp edges of GO nanowalls more easily damage the wall of Gram-positive bacteria [15], it should be taken into account that the thickness of the rigid peptidoglycan layer is much smaller in Gram-negative bacteria (2–3 layers compared to more than 10 layers in Gram-positive bacteria). Thus, we assume that the sharp nanowalls of GO-B quickly cut the liquid outer membrane layer and the thin peptidoglycan layer in Gram-negative bacteria, causing cytoplasm leakage. Moreover, in Gram-negative bacteria, the damage to the outer membrane itself inactivates these bacteria. Hence, the smaller GO-B nanowalls could cause damage and death to Gram-negative bacteria much faster than Gram-positive ones. The effect was readily observed in biofilms produced by Gram-negative bacteria, where GO-B inhibited the growth of biofilms better than GO-H. On the other hand, bigger nanosheets of GO-H reduced the total biofilm mass better than GO-B, most probably reducing the protective exopolysaccharide layer by bacteria forming biofilm, which could minimize biofilm viability and its resistance to environmental factors.

These features, i.e., nanosheet size and oxygen groups, translate into GO-B’s more potent antibacterial effect against Gram-negative bacteria, for which the MBC was ten times lower than for Gram-positive ones. Similarly, the applied concentrations of GO-B significantly influenced the percentage of dead cells within the biofilm formed by Gram-negative bacteria compared to that for GO-H. Thus, GO-B’s chemical features seem responsible for its better antibacterial activity than that of GO-H.

Most studies on the antibacterial biofilm activity of GO are conducted on commercially available GO produced by various modifications of Hummers’ method. Moreover, in the reports, GO is used in different concentrations, over different incubation times with bacteria, and with different species of bacteria. Therefore, until international standards are established for tests using specific GO concentrations and other experimental conditions, it will not be easy to assess clearly the antibacterial activity of GO. This is important because GO holds great promise for its use in medicine and may become essential in eradicating biofilms developing in chronic infections. In the meantime, more research is needed on the possibility of using GO to treat infections caused by antibiotic-resistant bacterial strains and associated biofilms.

However, our study has two significant limitations that make it challenging to analyze in detail the differences between the two types of graphene oxide we studied. First, more substantial and noisy peaks in XPS and FTIR result from measurement difficulties (proper flood and ion gun setting). Second, we did not perform Z-potential measurements, so the surface charges of both GOs are unknown. Therefore, GO-B and GO-H may differ in their surface charge, resulting in their different antibacterial activity.

Nevertheless, according to our research results, both graphene oxides showed differences in antibacterial activity, which gives rise to the assumption that the method of obtaining GO affects its properties. GO and its derivatives are widely used due to their physicochemical properties as construction materials and in medicine, e.g., as a drug carrier. As shown in many studies, GO-H is perfect for such applications. However, considering the advantage of GO-B as an antibacterial and anti-biofilm agent over GO-H, as shown in our studies, to receive better antibacterial activity and effectiveness as an anti-biofilm agent, e.g., in the hospital environment, GO should be obtained by Brodie’s method.

## 4. Conclusions

A comparison of GO obtained by Brodie’s and Hummers’ methods showed significant differences between both preparations. GO-B nanosheets were smaller, with more oxygen groups on the edges than GO-H. GO-B also showed much better bactericidal activity than GO-H, especially against Gram-negative bacteria and biofilms formed by them. Our results indicate that although obtaining GO-B is more complex, the resulting graphite oxidation product has an advantage over GO-H regarding its physicochemical and antibacterial properties.

## 5. Materials and Methods

### 5.1. Materials Used in the Study for Graphite Oxidation

Graphite powder (11 microns, 99% purity), synthetic graphite (conducting grade, 200 mesh, purity 99.9995%), and potassium chlorate (KClO_3_, purity >99%) were purchased from Alfa Aesar (Haverhill, MA, USA). Fuming nitric acid (HNO_3_, purity 98–100%) was purchased from Merck. Sulfuric acid (H_2_SO_4_, purity 98%, p.a.), hydrogen peroxide (H_2_O_2_, 30% *w*/*w* in H_2_O, p.a.), potassium permanganate (K_2_MnO4, p.a.), phosphorus pentoxide (P_2_O_5_, p.a.), and potassium persulfate (K_2_S_2_O_8_, p.a.) were purchased from Chempur (Piekary Slaskie, Poland). Hydrochloric acid (HCl, 35–38% *w*/*w*, p.a. basic) was purchased from Avantor Performance Materials Poland S.A. (formerly POCH, Gliwice, Poland).

### 5.2. Graphite Oxidation

**Figure 8:**
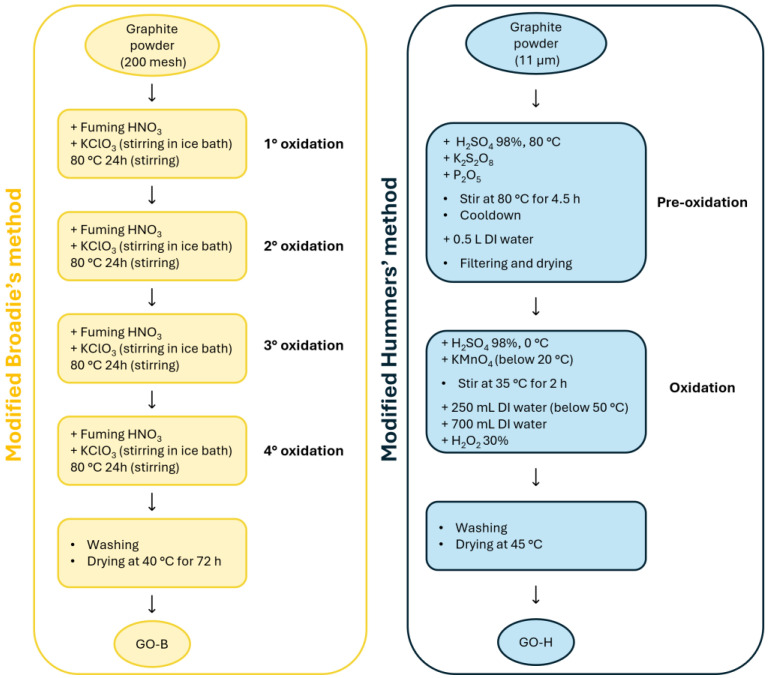
Schematic diagrams for the two methods employed for the preparation of GO-B (in yellow, **left**) and GO-H (in blue, **right**).

GO was synthesized from graphite powder using a modified Hummers’ method (GO-H) and a modified Brodie’s method (GO-B), both schematically reported in Figure 8. The GO-H sample was prepared following the procedure published by Xu et al. [47]. Briefly, graphite powder was put in hot, concentrated H_2_SO_4_ and pre-oxidized using K_2_S_2_O_8_ and P_2_O_5_, stirring the solution for 4.5 h on a hotplate. Then, the mixture was diluted with de-ionized water, filtered, and washed on a glass Büchner funnel. The pre-oxidized graphite was then oxidized using Hummers’ method. Pretreated graphite was added to cold, concentrated H_2_SO_4_, and then KMnO_4_ was added under stirring, using an ice bath to keep the temperature below 20 °C. After the addition, the solution was stirred for two hours and then diluted with de-ionized water, keeping the solution cold. The mixture was then stirred for another two hours and diluted again with 0.7 L of de-ionized water. After this last dilution, 20 mL of 30% H_2_O_2_ was added to the mixture, and the solution color changed from black/brown to bright greenish/yellow. Finally, the mixture was filtered and washed with 1:10 HCl aqueous solution and de-ionized water to remove the acid. The resulting solid was dried at 45 °C.

The GO-B sample was obtained by the modified Brodie’s method based on the work of Szabo et al. [48], by four-fold oxidation of synthetic graphite with KClO_3_ in fuming HNO_3_ with heating to 60–80 °C and mechanical stirring for 24–48 h. The obtained material (light yellow suspension) was washed with de-ionized water, then three times with 10% hydrochloric acid solution, and again with water until the filtrate reached pH = 7 [49,50]. Finally, the obtained GO was dried in a laboratory with forced air circulation for 72 h at 40 °C. GO-H and GO-B were dispersed in water using a high-power ultrasonic dispergator (20 kHz 900W UZDN-M-900-T Akademprylad, Ukraine) for two hours on 70% power. After the sonication step, both dispersions were homogeneous and stable. The dispersions were centrifuged twice at 8000 rpm for 10 min to remove bigger particles.

### 5.3. GO Characterization Techniques

Raman spectra were recorded in the 1000–1800 cm^−1^ range using a Renishaw InVia Raman spectrometer equipped with a confocal DM 2500 Leica optical microscope (Wotton-under-Edge, Great Britain, UK), a thermoelectrically cooled Charge Coupled Device (CCD) as a detector, and an argon laser operating at 514 nm, power 50% (~0.5 mW at sample surface), acquisition time 10 s and ten accumulations. Surface areas were measured using UV–vis spectroscopy with methylene blue (MB) as a probe, following the method described by Montes-Navajas et al. [51].

Absorption spectra were recorded using a BIOBASE BK-D590 double-beam scanning UV/Vis spectrophotometer (Jinan, China). The samples were first ground in a high-speed mill (20,000 rpm). After that, 5 mg of GO-H and GO-B were added to 10 mL of distilled and demineralized water, respectively, and the whole was dispersed with high-power ultrasonic dispergator (20 kHz 900W UZDN-M-900-T Akademprylad, Ukraine) for 30 min before measurement to make a stable dispersion of the composites in aqueous media.

Spectroscopic measurements were performed by placing 2 mL of water dispersion of each graphite oxide material in quartz cuvettes. A stock aqueous solution of MB (0.3 mg/mL) was prepared directly before measurements and used upon addition of increasing volumes (5 µL) of MB in each step (in the case of GO-B, after 100 µL of stock solution was added, the added portions were increased to 10 µL to reach 200 µL of added solution). The spectra were in the range of 400–800 nm. In this technique, by knowing the amount of used MB, which covered the surface of GO, surface areas of graphite oxides were calculated. XRD patterns were acquired by a PANalytical X’Pert pro-X-ray powder diffractometer (Panalytical, Eindhoven, The Netherlands) using nickel-filtered Cu Kα1 radiation operating at 40 keV and 30 mA. Infrared spectra were acquired using an infrared (MIR 400-4000) spectrometer FT-IR by Biorad 575C, with a 4000−30 cm^−1^ measurement range. Probes are measured in KBr pellets and directly from the probe surface with an ATR (Attenuated Total Reflectance) attachment device with a diamond crystal.

XPS measurements were performed on ThermoFisher Scientific Nexsa XPS. It has a monochromated, micro-focused, low-power Al K-Alpha X-ray source. Fifty scans were performed on C, O, and Survey.

TEM micrographs were acquired using Jeol’s JEM-F200 cold-FEG S/TEM at 200 kV. The samples were ground to a fine powder and suspended in ethanol. One drop of this suspension was put on a holey-carbon-coated copper grid of 300 mesh and left to air dry.

### 5.4. Antibacterial Efficacy of GO-B and GO-H

The plate colony counting method assessed the minimal inhibitory concentration (MIC) and minimal bactericidal concentration (MBC) of GO-B and GO-H in cell culture grade water. *Pseudomonas aeruginosa* (ATCC 27853), *Escherichia coli* (ATCC 25922), *Staphylococcus aureus* (ATCC 25923), and *Enterococcus faecalis* (ATCC 29212) reference strains were cultured overnight in the Mueller–Hinton broth (MHB). The cultures were centrifuged (16,000 rpm, 4 °C) and re-suspended in MHB to the optical density 1.5 *×* 10^6^ CFU (colony forming units)/mL corresponding to MacFarland standard 0.5. The GO-H and GO-B suspensions in water (1.0 mg/mL) were diluted ten-fold and plated at 10 µL into a 96-well plate. Then, 90 µL of bacterial suspensions in MHB were added into GO’s dilutions, mixed, and incubated for 4 h at 37 °C with shaking (80 rpm). Afterward, the MIC values were read. MIC reading is based on observation and spectrometer readings at 590 nm of the turbidity of the medium. The first well in which no turbidity of the culture is observed is considered the lowest inhibitory concentration (MIC). To determine the MBC value, 50 µL of the mixture was diluted in Mueller–Hinton agar (MHA) molten at 45 °C and poured onto sterile culture Petri dishes. Bacteria incubated for 4 h in MHB without GO-H and GO-B were negative controls. The plates were incubated overnight at 37 °C. The plate colony counting method was used to calculate GO-H and GO-B MBC values. Because *P. aeruginosa* and *E. faecalis* appeared in tiny colonies after 24 h, the incubation time was extended to 48 h. The assay was repeated three times in duplicate for every bacterial strain. The statistical significance of differences between means of results was determined by independent *t*-test and analysis of variance (one-way ANOVA with post hoc Tukey’s honestly significant difference) with *p* ≤ 0.05 considered statistically significant.

### 5.5. GO-B and GO-H Impact on Bacterial Biofilm Formation and Eradication

Biofilm formation by tested bacterial strains was assessed in the TSB with 0.5% glucose (TSB-G) for 48 h of incubation with shaking (400 rpm) at 37 °C. The biofilm formation was performed on round collagen-coated glass slides (rat tail collagen type I; 40 ug/cm^2^; Sigma-Aldrich, Darmstadt, Germany) for 24 h at 37 °C to mimic the host tissue environment. Overnight bacterial cultures in TSB were diluted 1:100 in a fresh TSB-G and inoculated into a 24-well plate with collagen-coated glass slides. GO-H and GO-B were added to the appropriate wells to final concentrations of 60 µg/mL for *E. coli* and *P. aeruginosa* and 120 µg/mL for *S. aureus* and *E. faecalis* (corresponding to double MIC values). Wells containing bacterial cultures in TBS-G without GO solutions were negative controls. Biofilm eradication was determined on biofilms of bacterial strains growing on collagen-coated glass slides for 48 h at 37 °C in TSB-G. The GO solutions were added to the wells with biofilms at concentrations used to assess biofilm production and incubated for an additional 24 h at 37 °C with shaking (400 rpm). After incubation, biofilms were washed carefully with sterile phosphate-buffered saline (PBS; pH 7.4) and stained with LIVE/DEAD assay (Sigma-Aldrich, Darmstadt, Germany) for 30 min in darkness, according to the manufacturer’s instruction. Then, the dyes were aspirated, and biofilms were washed once with PBS and transferred onto a glass slide to inspect under a fluorescent microscope. The experiment was repeated twice in duplicate. Imaging was performed on an Axio Inverted Observer fluorescence microscope 7 (Carl Zeiss, Erbach, Germany) equipped with an Orca Flash 40 camera (Hamamatsu, Hamamatsu-city, Japan) 10× objective Scale bar = 100 µm. A detailed computer-qualitative and quantitative analysis of the obtained pictures was performed by estimating the percentage of the area occupied by bacterial biofilm. Bacterial cell viability analysis was performed by calculating the fluorescence intensity of propidium iodide (Ex = 543 nm) and SYTO9 (Ex = 488 nm) dyes used in the LIVE/DEAD assay. Acquired images were processed and analyzed using Fiji/ImageJ software ver. 1.53c (NIH). First, maximum intensity projections (MIP) were obtained from stacks of images. The areas from binarized images were transferred onto original live and dead channel MIP images. The mean fluorescence intensities of all detected objects per field of view were calculated using ImageJ’s Analyze Particles function.

## Figures and Tables

**Table 1:** Description of the main modes in the FTIR spectra, according to Emiru et al. [25], and recorded for GO-H and GO-B samples in the study.

Signal [cm^−1^]	Description	GO-H	GO-B
~700–900	C-O in -C-O-C- (epoxy group)	704	687
	(stretching)		
~1050–1080	-C-O-C- (epoxy group), -C-O- (alkoxy groups)	1043	1051
	(stretching)		
~1220	-C=O, -C-OH (stretching)	–	1223
~1400	-O-H (deformation)	1378	1385
~1615	C=C (unoxidized graphitic domain),	1620	1620
	-OH, absorbed water (stretching)		
~1720	-COOH, -C=O (stretching)	–	1720
~2320	-C-H	2319	2319
~2800, ~2900	asymmetric and symmetric CH_2_	2800 sh.	2800 sh.
	Stretching	2900 sh.	2900 sh.
2500–3500	-O-H of carboxyl groups,	3352	3369
3400	-O-H, absorbed water		
	(stretching)		

**Table 2:** MIC and MBC values (µg/mL) for Gram-positive and Gram-negative bacterial strains were assessed after four hours of incubation with GO-B and GO-H in cell culture grade water.

Species	GO-B		GO-H	
	MIC	MBC	MIC	MBC
*S. aureus*	60	>1000	60	>1000
*E. faecalis*	60	1000	60	>1000
*P. aeruginosa*	30	100	30	100
*E. coli*	30	100	30	100

## Data Availability

The datasets generated during and/or analyzed during the current study are available from the corresponding author upon reasonable request.
